# Metabolic changes in psoriatic skin under topical corticosteroid treatment

**DOI:** 10.1186/1471-5945-13-8

**Published:** 2013-08-14

**Authors:** Beathe Sitter, Margareta Karin Johnsson, Jostein Halgunset, Tone Frost Bathen

**Affiliations:** 1 Department of Technology, Sør Trøndelag University College, 7004 Trondheim, Norway; 2 Department of Dermatology, St. Olavs Hospital HF, Trondheim University Hospital, Trondheim, Norway; 3 Department of Cancer Research and Molecular Medicine, Norwegian University of Science and Technology, Trondheim, Norway; 4 Department of Laboratory Medicine, Children’s and Women’s Health (LKB), Norwegian University of Science and Technology, Trondheim, Norway; 5 Department of Pathology and Medical Genetics, St. Olavs Hospital HF, Trondheim University Hospital, Trondheim, Norway; 6 Department of Circulation and Medical Imaging, Norwegian University of Science and Technology, Trondheim, Norway

**Keywords:** Tissue, Metabolites, Corticosteroids, Psoriasis treatment, MR spectroscopy

## Abstract

**Background:**

MR spectroscopy of intact biopsies can provide a metabolic snapshot of the investigated tissue. The aim of the present study was to explore the metabolic pattern of uninvolved skin, psoriatic skin and corticosteroid treated psoriatic skin.

**Methods:**

The three types of skin biopsy samples were excised from patients with psoriasis (N = 10). Lesions were evaluated clinically, and tissue biopsies were excised and analyzed by one-dimensional ^1^H MR spectroscopy. Relative levels were calculated for nine tissue metabolites. Subsequently, relative amounts of epidermis, dermis and subcutaneous tissue were scored by histopathological evaluation of HES stained sections.

**Results:**

Seven out of 10 patients experienced at least 40% reduction in clinical score after corticosteroid treatment. Tissue biopsies from psoriatic skin contained lower levels of the metabolites *myo*-inositol and glucose, and higher levels of choline and taurine compared to uninvolved skin. In corticosteroid treated psoriatic skin, tissue levels of glucose, *myo*-inositol, GPC and glycine were increased, whereas choline was reduced, in patients with good therapeutic effect. These tissue levels are becoming more similar to metabolite levels in uninvolved skin.

**Conclusion:**

This MR method demonstrates that metabolism in psoriatic skin becomes similar to that of uninvolved skin after effective corticosteroid treatment. MR profiling of skin lesions reflect metabolic alterations related to pathogenesis and treatment effects.

## Background

Psoriasis is a common immune-mediated disease that affects the skin and joints. The cause of the disease remains unknown. Many patients have a genetic predisposition. The disease affects around 2–3% of the population worldwide. Clinically, psoriatic plaques are characterized by sharply demarcated erythematous lesions with thick silvery scales, often distributed in a symmetrical pattern. Histopathologically there is hyperproliferation of epidermal cells and an inflammatory cell infiltrate [1]. There is increasing awareness that psoriasis is a multisystem affection with substantial comorbidity, particularly of cardiovascular diseases and metabolic syndrome [2]. The course is that of a chronic, relapsing disease which requires long term treatment. Various topical and systemic treatment options exist for psoriatic lesions. Topical corticosteroids remain the cornerstone, either used as monotherapy or in combination with other treatment modalities. These agents exert anti-inflammatory and immunosuppressive effects by stimulation or inhibition of the genes involved in inflammatory pathways, including inhibition of cytokine production and reduction of such mediators of inflammation as prostaglandins and leucotrienes, inhibition of T-cell proliferation and T-cell dependent immunity, and suppression of fibroblast and endothelial cell functions [3,4]. Corticosteroids also have anti-proliferative effects, by delaying the onset of DNA synthesis and decreasing the mitotic rate [5].

Molecular studies of outbreak and healing of psoriatic lesions can provide insight in the underlying biological processes. Genome wide association scans (GWAS) have identified genetic susceptibility factors [6], and molecular analysis have revealed associations of psoriasis with specific molecular pathways [7,8]. Detailed molecular characterization of autoimmune diseases can provide information about mechanisms involved in disease progression and action of drugs, and also provide biomarkers to predict and monitor disease course. Cellular enzymatic processes involve small molecular metabolites as substrates, intermediates and end products, and such metabolites are crucial in energy turnover and membrane synthesis. Metabolic studies have been applied in numerous biomedical settings [9], and for instance metabolic characterization of cancerous tissue is expected to contribute to a more detailed tumor portrait by defining specific fingerprints reflecting diagnostic status or predicting therapeutic response [10]. Magnetic resonance spectroscopy (MRS) analysis of intact tissue specimens can provide a detailed description of the biochemical composition of the tissue, using so-called high resolution (HR) magic angle spinning (MAS) MRS. This technology requires a minimum of preparation of samples, and detailed biochemical information can be obtained from small specimens (typically 20 mg). Multiple cellular metabolites can be measured simultaneously, and the sample is kept intact for subsequent analysis by other techniques.

The purposes of the present study were to characterize the metabolic patterns of intact uninvolved and affected skin in psoriasis patients and to monitor the biochemical changes in psoriatic skin accompanying corticosteroid treatment. Ten patients were included, three biopsy samples being excised from each: uninvolved skin, psoriatic skin, and corticosteroid treated psoriatic skin, respectively. All biopsy samples were investigated by MAS MRS, and the resulting spectra were further analyzed by peak area calculations to obtain relative measures of tissue metabolite contents.

## Methods

### Subjects

Ten patients with stable light to moderate plaque psoriasis volunteered to participate in the study. Eight of the patients were men and two were women (not pregnant or nursing) with a median age of 52 (range 28–75) years. None of the patients used systemic treatment for psoriasis. Three patients were on systemic medication for non-dermatological reasons: irbesartan (hypertension) and terbinafine (tinea unguium), aspirin and pravastatin (hypercholesterolemia) and amlodipine (hypertension). The Regional Committee for Medical and Health Research Ethics, Central Norway approved the study protocol, and all patients signed a written informed consent form.

### Study design

Two symmetrical psoriatic lesions were chosen for each patient. The psoriatic lesions were localized at elbows (n = 3), knees (n = 3), upper back (n = 1), hips (n = 1), flanks (n = 1) and buttocks (n = 1). After at least two weeks of treatment with only emollient (Locobase®, Yamanouchi), one plaque was assigned for continued treatment with emollient. The other chosen psoriatic lesion was treated once daily (evening time) with the very potent corticosteroid clobetasol propionate ointment 0,05% (Dermovate®, GlaxoSmtihKline). In addition, the emollient was used both on the lesion treated with corticosteroid and the control lesion according to needs. Both psoriatic lesions were evaluated clinically before the start of the treatment and after four weeks of treatment. The severity of scaling, erythema and infiltration of the lesions was scored on a scale from 0–4 for each parameter (0 absent and 4 severe). After four weeks, three punch biopsies (4 mm) were taken after local anaesthesia with lidocaine with epinephrine: from uninvolved skin, from psoriatic skin and from corticosteroid treated psoriatic skin in the same body area.

### Sample treatment

The punch biopsy samples were put in a cryo-tube and frozen in liquid nitrogen (-195.8°C) within one minute after tissue resection, and further stored in liquid nitrogen until MR analysis. Samples weighed 21.9 mg on average (range from 11.6 to 35.7 mg).

### MR spectroscopy

The MR experiments were performed as previously described [11]. Briefly, samples were thawed on an ice-block to provide a cold environment, and transferred to a 4 mm MAS rotor (total sample volume 50 μL) containing 40 μL phosphate buffered saline with TSP (1 mM). The rotor was thereafter placed in a Bruker Avance DRX600 spectrometer equipped with a ^1^H/^13^C MAS probe with gradients (Bruker BioSpin GmbH, Germany). During signal acquisition, which started within 42 minutes in average after sample thawing (maximum 1 hour and 35 minutes), the samples were spun at 5 kHz and kept at 4°C. One-dimensional ^1^H spectra were recorded, using a spin-echo sequence which suppresses broad peaks and the water signal. The resulting spectra are highly resolved, with relatively enhanced signals from small metabolites. Spectral assignments were performed based on metabolite appearances in previously recorded MR spectra of intact human tissue [12,13] and in MR spectra of extracts from skin tissue [14]. Totally 17 metabolites were assigned.

### Analysis of MR spectra

The spectral region 4.7 to 3.0 ppm was used for peak area calculations, which was performed using the curve fitting program PeakFit version 4 (SeaSolve Inc, USA (MA)), by combined Lorenzian and Gaussian functions (Voigt area) for curve area estimation. Areas were calculated for the nine peaks arising from glucose, lactate, *myo*-inositol, glycine, taurine, glycerophosphocholine (GPC), phosphocholine (PCho), choline and creatine. The program uses a least squares function to optimize the fit to the real spectrum, and the correlation factor which describes the goodness of fit was better than 0.95 for all area calculations. To obtain a semi-quantitative measure for each metabolite, its peak area was normalized to the total peak area of all nine metabolites for every spectrum. Kruskal-Wallis multiple sample analysis was applied for paired comparisons of metabolite content in the three types of skin samples. Differences in tissue metabolites between corticosteroid treated and untreated psoriatic skin were calculated for all patients. Metabolic changes ascribed to corticosteroid treatment were compared between the group with poor (N = 3) and good (N = 7) clinical effect using Mann–Whitney significance test. Statistical analyses were performed using SPSS (SPSS 16, SPSS Inc.).

### Histopathology

Tissue samples were stored in liquid nitrogen for 20 months after MAS MRS analysis. For histological evaluation, samples were thawed and immersed in 4% buffered formaldehyde fixative solution for 24 hours, followed by embedment in paraffin. From each block, one 5 μm tissue section was cut and stained with haematoxilin, erythrosine and saffron (HES). The microscopic sections were photographed with a digital camera, and the relative amounts of epidermis, dermis and subcutaneous tissue were determined by point counting [15]. Briefly, the micrographs were overlaid with a randomly positioned point grid, and the number of points falling on each of the three tissue components was counted, considering the stratum corneum as part of the epidermis. The relative number of points falling on one particular component was taken as an estimate of the section area occupied by the respective tissue element. The area fraction thus obtained is an unbiased estimate of the corresponding volume fraction in the tissue sample, provided the section is chosen randomly.

## Results

### Patients

All patients experienced a reduced degree of psoriatic affection after four weeks of treatment with corticosteroid ointment. Seven of the patients experienced at least 40% reduction of the clinical score of the skin, of which four patients had almost complete normalization of the skin (score grade 1 for erythema and infiltration, no scaling). Three patients had 40% or less reduction of clinical skin scoring, and were considered to have poor effect of the corticosteroid treatment. Concerning the untreated psoriatic lesions, five of the patients showed no change over four weeks, three experienced less scaling after application of emollient whereas in two patients a worsening was noted.

### Histology

All samples could be evaluated with respect to tissue composition after MR analysis and long-term storage in liquid nitrogen. Epidermis was thicker in psoriasis lesions, and comprised a significantly larger fraction of the biopsies both in untreated (12%) and corticosteroid treated (9%) skin than in the uninvolved skin (3%) (p < 0.05, ANOVA). All patients but one had lower epidermal fraction in corticosteroid treated than in untreated psoriatic skin, with about 40% reduction in epithelial thickness. The one patient with an increased thickness of epidermis after corticosteroid treatment was also clinically scored as showing poor response to treatment.

### Metabolites

**Figure 1:**
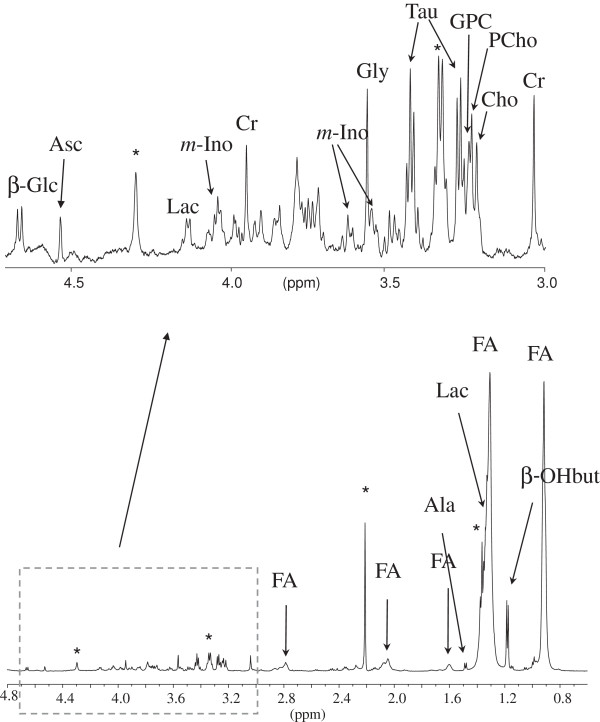
**HR MAS spectrum of psoriatic skin biopsy (not treated).** The spectral region 4.8 to 0.3 ppm is shown (lower part) with the expanded region 4.8 to 3.0 ppm above. Abbreviations used: β-Glc: β-Glucose, Asc: Ascorbate, Lac: Lactate, m-Ino: *myo*-Inositol, Cr: Creatine, Gly: Glycine, Tau: Taurine, GPC: Glycerophosphocholine, PCho: Phosphocholine, Cho: Choline FA: Fatty Acids, Ala: Alanine and βOH-But: β-hydroxybutyrate. *: Denotes signals from lidocaine.

**Figure 2:**
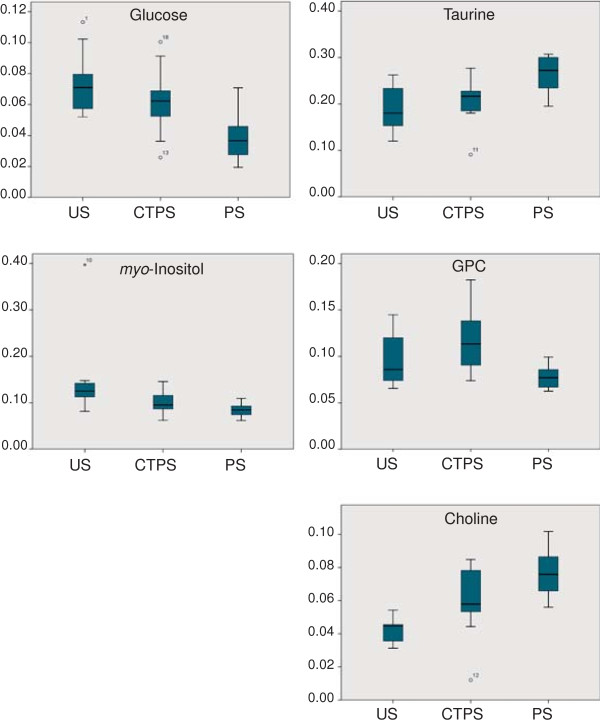
**Box-plot of relative levels of tissue metabolites in uninvolved skin (US), corticosteroid treated psoriatic skin (CTPS) and psoriatic skin (PS).** Only metabolites found to be differently expressed in different types of samples by statistical analysis (p < 0.05, Kruskal-Wallis) are shown: glucose, *myo*-inositol, taurine, glycerophosphocholine and choline.

The MR spectra of the skin samples showed signals from numerous small molecular weight metabolites and lipids (Figure 1). The nine metabolites were detectable in all spectra, and were identified as cell building blocks (amino acids and choline compounds), osmolytes (taurine) and metabolites involved in energy consumption (glucose and lactate). Peak areas of the nine selected metabolites could be calculated for all samples. In addition, the anesthetic lidocaine contributed significantly to most spectra, giving rise to a total of seven peaks. Statistical analysis showed that the tissue content of glucose, *myo*-inositol, taurine, GPC and choline were different in the three types of sample (p < 0.05, Kruskal-Walis) (Figure 2). The levels of *myo*-inositol and glucose were highest in uninvolved skin and lowest in psoriatic skin, whereas those of taurine and choline were highest in psoriatic skin and lowest in uninvolved skin. The levels of GPC were highest in corticosteroid treated skin and lowest in psoriatic skin. We observed no differences in the tissue levels of creatine, glycine, lactate or phosphocholine between the three types of sample.

**Figure 3:**
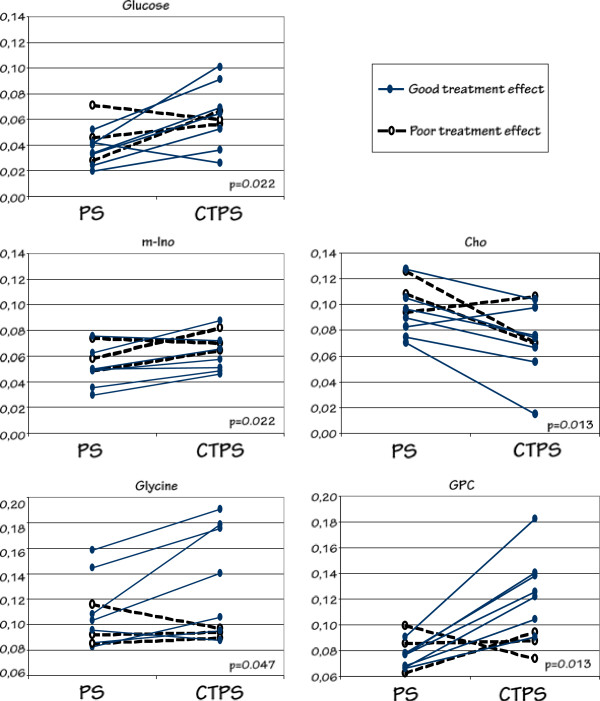
Differences in tissue metabolic levels of glucose, myo-inositol, glycine, GPC and choline between psoriatic skin (PS) and corticosteroid treated psoriatic skin (CTPS), discriminating patients with good effect of corticosteroid treatment (n = 7) and patients with poor effect of corticosteroid treatment (n = 3).

Seven of the patients showed a good clinical effect of topical corticosteroid treatment (at least 40% reduction of clinical score), whereas three patients had poor effect (less than 40% reduction of clinical score). We found metabolic changes that were different in these two patient groups for five of the metabolites (p < 0.05, Mann–Whitney) (Figure 3). In skin samples from patients with good treatment results, glucose, *myo*-inositol, GPC and glycine increased with treatment, whereas choline decreased.

## Discussion

Four weeks of anti-psoriatic treatment with topical corticosteroids led to clinical improvements, as expected. This was recorded as reductions in the clinical score for erythema, infiltration and scaling. We also observed by histopathology that the relative thickness of the epidermis was reduced in nine out of 10 patients as an effect of corticosteroid treatment.

The spectral quality was partly influenced by the heterogeneity of the skin biopsies and by signals from the local anesthetic. MR spectroscopy of intact skin biopsies provided semi-quantitative information about tissue metabolites. It was not possible to perform absolute quantification, as this would require T2 measurements to allow for T2 correction. The MR acquisition protocol could thus not provide an absolute quantification of tissue metabolites, but the relative quantification enabled inter-sample comparisons.

We found that psoriatic skin had significantly lower levels of *myo*-inositol and glucose, and higher levels of choline than uninvolved skin. These differences between affected and healthy tissue of psoriatic patients are analogous to the differences found between affected and healthy tissue in cancer patients [10]. Both psoriasis and cancer are characterized by high cellular proliferation rates. Similarities in metabolic profiles are thus expected, in particular higher availability of nutrients for the synthesis of new biomass. Lower levels of glucose are presumably due to high glucose turnover in rapidly proliferating cells, whereas increased choline levels are associated with the need of cellular building blocks [10]. In a previous study by Kim *et al.*[14], perchloric acid extracts of skin biopsy samples from psoriasis plaques, malignant melanomas and control skin were analyzed with MR spectroscopy and GC/MS. Several metabolic ratios and concentrations were altered in psoriasis plaques compared to uninvolved psoriatic skin. The majority of metabolite concentrations were higher in psoriasis plaque. For metabolites analyzed in our study, Kim *et al.*[14] reported higher creatine-to-glycine ratio, higher lactate and lower glycine in psoriatic plaque than in non-involved psoriatic skin. The reduced glycine in steroid treated skin from responders (Figure 3) resembles the lower glycine in non-involved skin reported by Kim *et al*. No other similar metabolic findings were observed, possibly due to different approaches in metabolite analysis.

We observed increased levels of glucose and *myo*-inositol, and decreased levels of choline in steroid treated skin from responders, which indicate that the metabolic profile approaches that of uninvolved skin when treatment is effective. This metabolic change probably reflects the anti-proliferative effect of steroids, which leads to a decreased demand of nutrients for biomass synthesis. The decreased choline levels can also reflect the anti-inflammatory effect of corticosteroid treatment, as choline is known to accumulate in inflammatory processes [16]. Thus, the metabolic profile of tissue can be a molecular indicator of treatment effects and provide insight into specific pathways of drug action. There is an increased understanding of changes in molecular and cellular processes in psoriasis, and the roles and functions of several macromolecules are established. The method applied in this study can portray the small organic molecules that are substrates, intermediates and end products in cellular processes. Their function and role in psoriasis is not established. In order to understand how drugs affect metabolic pathways, tissue metabolic analysis should be combined with molecular profiling [17].

This study was performed on a small number of patients (N = 10), where three of the patients were receiving systematic treatment for other conditions. These ongoing, systematic treatments are unlikely to influence the observed metabolic patterns and alterations in the skin. However, a larger population without patients receiving systematic treatments will rule out possible biasing and provide more robust data. This study demonstrates the potential value of metabolic profiling as a provider of information complementary to clinical and molecular evaluation, including information which may shed new light on the changes occurring with treatment. By modifying the experimental procedures we may obtain absolute quantitative measures of tissue metabolites [18]. Furthermore, metabolic profiling can be combined with subsequent analysis of gene expression profiles [17,19], thus enabling detailed and complex information on several molecular levels. Although demonstrated for topical corticosteroid treatment, metabolic profiling of psoriatic lesions can also be applied in a diverse range of psoriasis treatment regimens, providing increased understanding of the processes of pathogenesis and their reversal due to treatment. MR spectroscopy of skin biopsies may also be utilized to obtain metabolic status of other skin conditions.

## Conclusion

This study demonstrated detectable differences in tissue metabolites between uninvolved skin, psoriatic skin and corticosteroid treated psoriatic skin. We also found that metabolic differences induced by corticosteroid treatment were related to the actual therapeutic effect. The application of MR spectroscopy in dermatological research provides information about tissue metabolites, thus presenting a novel approach for studies of pathogenesis and treatment effects in the skin.

## Competing interests

None of the authors have any financial or non-financial competing interests in the findings from this manuscript.

## Authors’ contributions

All authors have significantly contributed to the manuscript. All read and approved the final manuscript. BS and MKJ have designed the study. BS, MKJ and JH have conducted acquisition of data. BS and TFB have performed data analysis and interpretation. BS, MKJ, JH and TFB have all been involved in drafting the manuscript and revising it critically for important intellectual content.

## Pre-publication history

The pre-publication history for this paper can be accessed here:

http://www.biomedcentral.com/1471-5945/13/8/prepub
